# Transcatheter therapies for secondary mitral regurgitation in advanced heart failure: what are we aiming for?

**DOI:** 10.1007/s10741-021-10148-z

**Published:** 2021-07-22

**Authors:** Andrea Scotti, Andrea Munafò, Alberto Margonato, Cosmo Godino

**Affiliations:** 1 Department of Cardiac Thoracic Vascular Sciences and Public Health, University of Padua Medical School, Via Giustiniani 2, 35128 Padua, Italy; 2 Division of Cardiology, IRCCS Policlinico San Matteo Foundation, Pavia, Italy; 3 Division of Cardiology, San Raffaele Scientific Institute, Milan, Italy

**Keywords:** Mitral regurgitation, Advanced heart failure, Transcatheter mitral valve repair, Transcatheter mitral valve replacement, Heart transplantation

## Abstract

A severe secondary mitral regurgitation (SMR) can be found in a significant portion of patients affected by advanced heart failure (AHF). Conventional therapies (optimal medical therapy, devices, surgery) present restricted clinical efficacy in this stage of the left ventricle disease which is burdened by high mortality and morbidity rates. Although the treatment of choice is represented by heart transplantation (HTx), there is an unmet need related to the limited supply of donor hearts (as opposed to the growing prevalence of AHF) and the low eligibility of highly symptomatic patients. In case of concomitant severe SMR, transcatheter mitral valve therapies (repair and replacement) may play a crucial role in this setting. While a direct prognostic improvement after correction of SMR has yet to be proved, AHF patients can benefit from the following: hemodynamic stabilization, symptomatic relief, normalization of pulmonary arterial pressures, and reduction in hospitalizations for acute heart failure. Obtaining these results may lead to the clinical consequences of reaching the HTx in good enough clinical status (bridge to heart transplantation), becoming eligible for the HTx (bridge to HTx candidacy), and being delisted for clinical improvement (bridge to recovery). Therefore, achieving traditional secondary endpoints in patients with AHF and SMR can translate into significant clinical implications.

## Introduction

The burden of heart valve diseases represents a major public health problem. In the developed countries, mitral regurgitation is the most common valve disorder with a prevalence increasing with age [1]. Particularly, due to the increased number of patients with left sided heart failure, secondary mitral regurgitation (SMR), a common phenomenon in this scenario, has become the most frequent form.

The latter constitutes both a diagnostic and a therapeutic challenge. Up to 24% of patients with SMR are affected by heart failure due to left ventricular (LV) dysfunction leading to an underestimation of the mitral valve disease itself [2, 3]. Since LV disease is the most important prognostic factor, guideline-directed medical therapies (GDMT) for heart failure play a major role for these patients. However, SMR is not a merely bystander but contributes to worsening survival and LV function [4–6]. This vicious cycle may lead to a stage of advanced heart failure (AHF) where patients no longer respond to conventional therapies. While the correction of SMR has yet to prove its effectiveness in terms of survival, it has been shown how it can translate into significant clinical implications for AHF patients.

## Advanced heart failure

Chronic heart failure is a common condition among adults, with an increasing prevalence because of the aging of the population and improved cardiovascular treatments (especially for acute events). Although it is a heterogeneous syndrome, all etiologies share a final common pathway with progressive worsening of symptoms and cardiac function. The final stage when conventional therapies (GDMT, implanted devices, surgery) are no more effective is represented by the AHF, clinically diagnosed on the basis of patient’s symptoms, cardiac dysfunction, functional capacity indices, and prognostic markers. The low incidence and the evolving definitions of AHF make it difficult to estimate its prevalence. Data from ADHERE (Acute Decompensated Heart Failure) national registry of ~ 23,000 hospitalized patients report a prevalence of AHF close to 5% [7]. Since the AHF is burdened by high mortality and morbidity rates, early recognition of this stage of the disease is of paramount importance. Indeed, the heart failure association of the European Society of Cardiology stresses the importance of a timely referral of patients with AHF to tertiary hub centers where the broad spectrum of advanced therapies can be offered [8]. The eligibility for such specialized care can be verified with the useful mnemonic “I Need Help” consisting of inotropes, New York Heart Association (NYHA) class II/III or elevated natriuretic peptides, renal or liver dysfunction, very low ejection fraction (< 20%), recurrent appropriate defibrillator shocks, hospitalizations for heart failure in the previous 12 months, edema or increasing diuretic doses, low blood pressure (systolic < 90 to 100 mmHg), and inability to up-titrate or maintain prognostic drugs [9].

### Therapies for advanced heart failure

The treatment of choice for most patients with AHF is represented by heart transplantation (HTx). To date, the complications related to infectious diseases, immunosuppression, and donor/recipient selection have been significantly reduced. As a result, survival, quality of life, and exercise tolerance increased. The main limitations of this treatment consist in the limited availability of donor hearts and the poor eligibility of patients who are often in such a compromised stage that no benefit can be expected. While the number of transplants appears to have reached a plateau, the HTx waiting lists are persistently expanding. Prolonged (over 12 months) times on waiting lists are associated with mortality rates ranging between 13% at 1 year and 19% up to 3 years [10].

Long-term mechanical circulatory support (MCS) obtained through left ventricular assist device (LVAD) implantation represents a successfully therapeutic solution for this population. The Risk Assessment and Comparative Effectiveness of Left Ventricular Assist Device and Medical Management in Ambulatory Heart Failure Patients (ROADMAP) study provided the risk–benefit ratio for a shared decision on elective LVAD therapy for AHF. Higher survival with improved functional status, improved quality of life, and reduced depression favored LVAD over optimal medical therapy; on the other hand, LVAD implantation caused more hospitalizations for heart failure and major adverse events (bleedings, arrhythmias) that reduced after the first year of follow-up [11].

LVAD placement is now indicated for AHF patients (1) not eligible for HTx (LVAD as destination therapy, actually the main application worldwide), (2) with reversible contraindications to HTx (e.g., obesity, pulmonary arterial hypertension, or renal failure) whose resolution could establish the feasibility of HTx (bridge to candidacy, *Class IIb, Level of Evidence: C*) [12], or (3) with the possibility of cardiac function recovery (bridge to recovery). Moreover, (4) AHF patients who are still on the HTx waiting list, could undergo LVAD implantation in order to keep them alive without worsening hemodynamics until a donor organ becomes available (bridge to HTx) [13, 14].

In the wide spectrum of AHF patients, LVAD is still reserved for those with an extremely advanced clinical condition (identified as patients with an INTERMACS profile of 1 to 3) [14]. Besides, LVAD implantation has an inherent risk of serious adverse events, whose perceived impact has even more value in patients with prohibitive operative risk, limited life expectancy, and severe psychosocial limitations. As a result, a significant portion of them is not eligible for MCS or refuses this therapy for several personal reasons.

Finally, according to the fluctuating and progressive history of the disease, AHF patients might require short term MCS due to sudden worsening of their clinical condition. Among the percutaneous options, there are four established devices including Impella (Abiomed, Danvers, MA), TandemHeart (CardiacAssist, Pittsburgh, PA), extracorporeal membrane oxygenation, and intra-aortic balloon pump. All of them might guarantee hemodynamics and end-organ perfusion stabilization, allowing patients to be eligible for HTx or long-term MCS.

### Secondary mitral regurgitation

Hemodynamically significant SMR is a common finding in patients affected by AHF: severe or moderate-severe SMR in about 15% and moderate or worse SMR in about 40% [15]. The severity of SMR is associated with the degree of LV dilatation, systolic/diastolic LV dysfunction, and pulmonary hypertension, resulting in an exacerbation of heart failure symptoms.

In heart failure with reduced ejection fraction, SMR usually develops as a “ventricular-secondary” mechanism: a competent coaptation of mitral valve leaflets is impaired by mitral annulus dilation and papillary muscles displacement due to LV dilatation. The common coexistence of left atrial dilatation and/or atrial fibrillation results in the elevation of atrial pressures and dilatation of mitral annulus that worsen the effects and prognosis of SMR with an “atrial-secondary” mechanism [16, 17]. This is the rationale for the improvement of SMR after GDMT for heart failure (*ventricular-secondary*) and rhythm/rate control for atrial fibrillation (*atrial-secondary*).

When AHF symptoms and SMR severity no longer respond to conventional heart failure therapies, it is likely that AHF has evolved to valvular-AHF. In this context, mitral valve intervention may play a key role in patients’ treatment by ending this vicious cycle. Obtaining a hemodynamic stabilization could enable the up-titration of GDMT and provide more time to reach advanced therapies. Considering the association of AHF and high/prohibitive surgical risk, the reduction of SMR could be addressed in most AHF patients with a transcatheter solution [18, 19].

## Transcatheter mitral valve repair

**Fig. 1:**
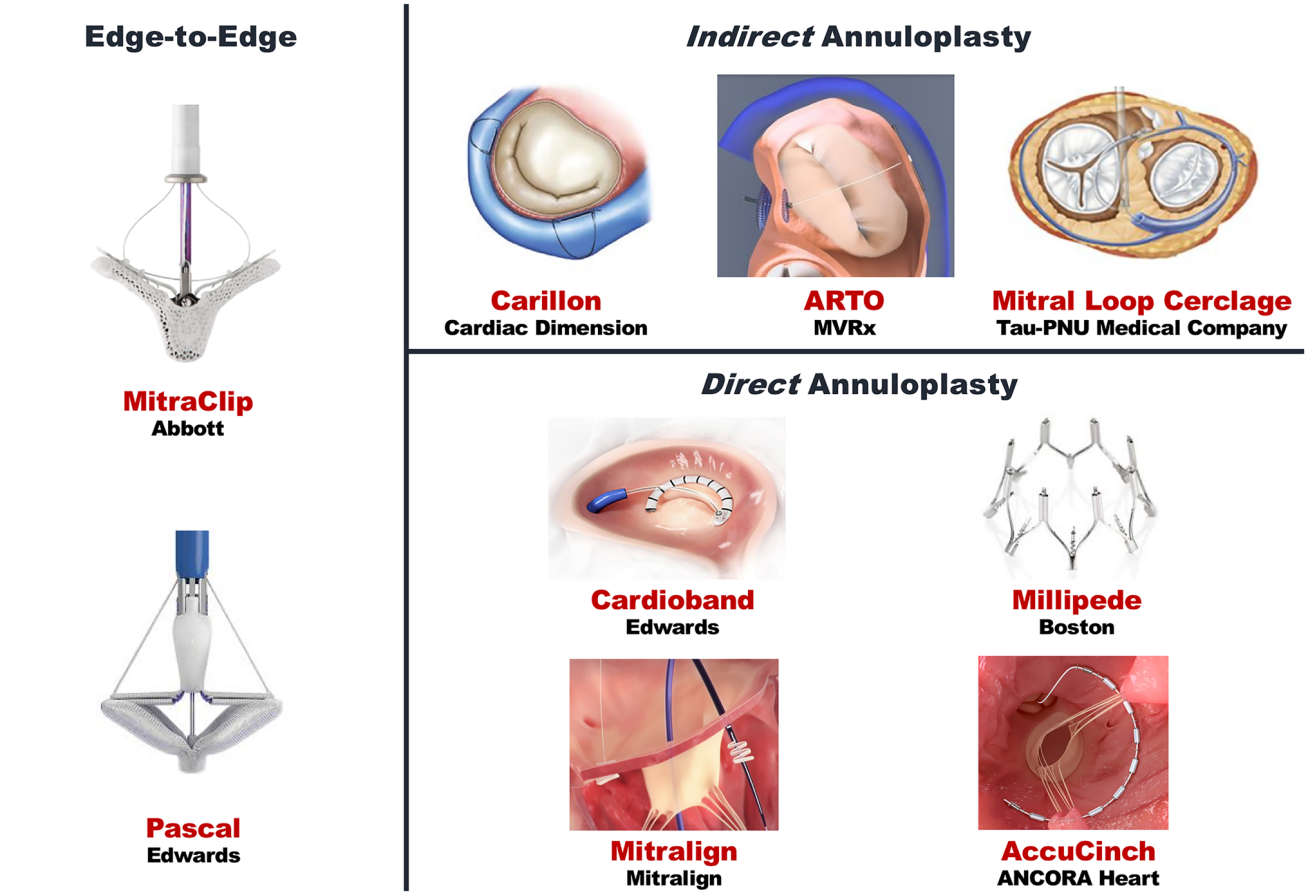
Transcatheter mitral valve repair devices. Reproduced from Vessel Plus 2021;5:6 [20]

The available transcatheter mitral valve repair (TMVR) techniques for SMR (Fig. 1) [20] can be classified as follows:

- Edge-to-edge repair = MitraClip, PASCAL
- Percutaneous annuloplasty = AccuCinch, ARTO, Carillon, Cardioband, Millipede, Mitral Loop Cerclage, Mitralign

The MitraClip (Abbott Laboratories, Menlo Park, CA, USA) device is a clip that reproduces the surgical Alfieri-stitch by grasping the mitral valve leaflets through its arms and providing a more competent coaptation. The PASCAL (Edwards Lifesciences, Irvine, CA) system adopts the same rationale of the MitraClip with the addition of a 10-mm central spacer within the regurgitant office to reduce the tension on the valve leaflets caused by the traction paddles. Favorable features are represented by a grasping length > 10 mm, a coaptation length ≥ 2 mm, and a coaptation depth ≤ 11 mm [21]. Suboptimal results may be expected in the presence of leaflets’ perforations, calcifications on the grasping zone, or deep cleft-like indentations. Pre-procedural mitral valve area ≤ 3 cm^2^ and mean mitral valve gradient > 4 mmHg need to be excluded to prevent a procedural failure related to intractably high transmitral gradients [22].

### Percutaneous annuloplasty

 is the solution to mitral annulus dilatation, whose narrowing allows to approximate the valve leaflets and reduce the orifice area. The *direct* approach (Cardioband, Millipede, Mitralign, AccuCinch) can be performed by a transseptal or retrograde LV access and consists of implanting a variable number of anchors around the mitral annulus and tether them with the delivery system. *Indirect* annuloplasty devices (Carillon, ARTO, Mitral Loop Cerclage Catheter System) promote a positive remodeling by applying a traction force on the coronary sinus. The main limitations of these procedures rely on their complexity and long duration, unfavorable anatomy of the coronary sinus and its branch veins, and the risk of coronary compression. An angio-CT is mandatory in the screening phase to exclude the presence of calcifications, the risky proximity of the left circumflex artery, and tricky anatomies. When suboptimal results are expected, percutaneous annuloplasty can be performed as a complimentary technique to edge-to-edge repair [23].

### Clinical evidence

**Fig. 2:**
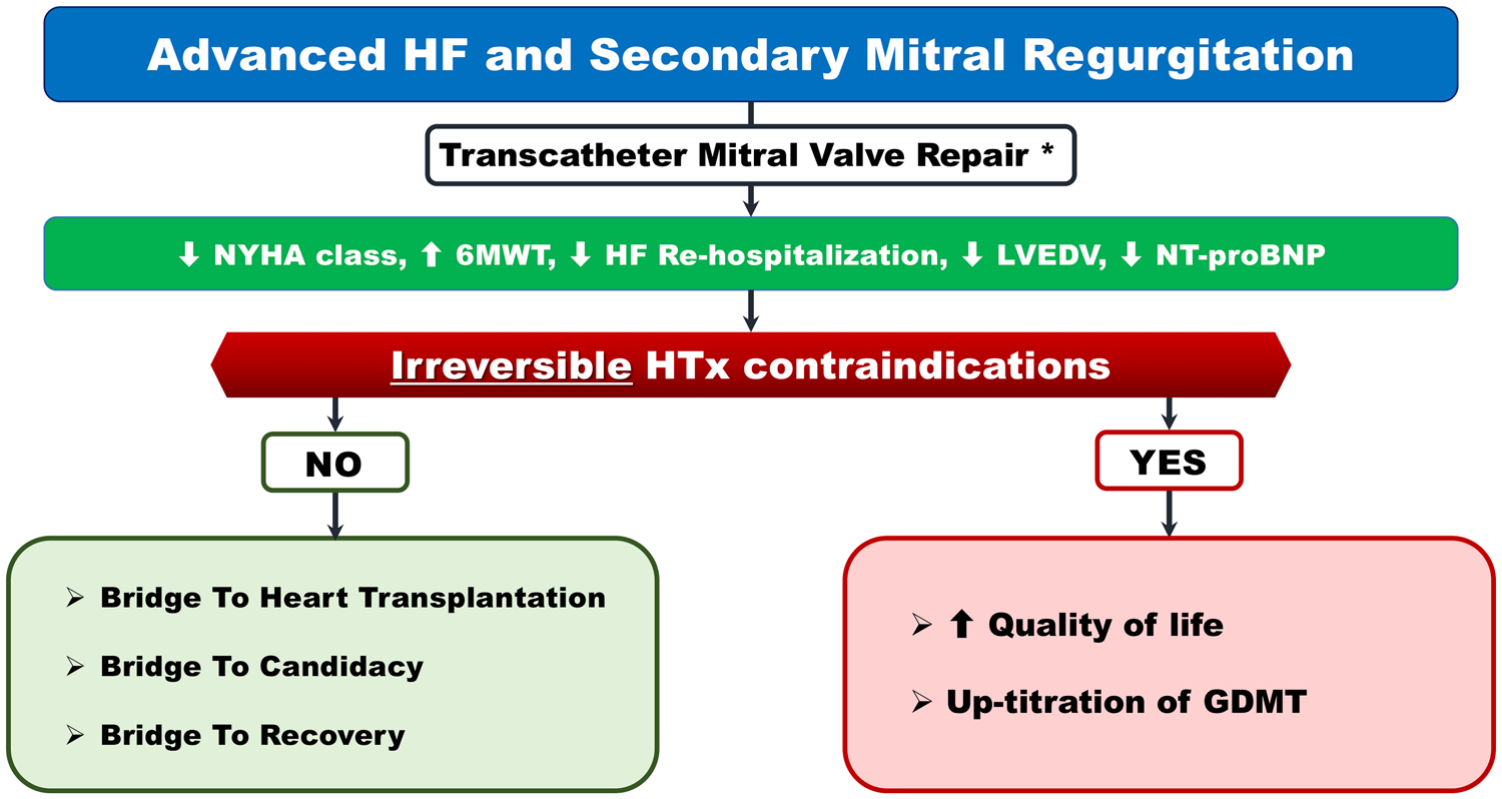
Expected outcomes after transcatheter mitral valve repair in patients with advanced heart failure and secondary mitral regurgitation. *Published evidences on MitraClip implantation. GDMT, guideline directed medical therapy; HF, heart failure; HTx heart transplantation; LVEDV, left ventricular end diastolic volume; NT-proBNP, N-terminal pro-brain natriuretic peptide; NYHA, New York Heart Association; 6MWT, 6-min walk distance

The reduction of SMR in AHF patients provides clinical and hemodynamic benefits that result in less severe symptoms, fewer heart failure hospitalizations, and, in some cases, LV remodeling (Fig. 2). The clinical implications of these outcomes rely on the presence of irreversible contraindications to HTx. If this condition occurs, the purpose of SMR reduction consists in providing an improved quality of life and enabling the up-titration of GDMT. In AHF patients who are potentially eligible for HTx (absent or reversible contraindications), the rationale for treating SMR can be classified into the following strategies:

- Bridge to heart transplantation (BTT)
- Bridge to HTx candidacy (BTC)

- Bridge to recovery (BTR)

Of all the TMVR systems, the MitraClip is the only one that has strong evidence for clinical efficacy, with the other devices having limited system numbers or being no longer available. In 2011, Franzen and colleagues were the first to report the feasibility and effectiveness of MitraClip implantation in their cohort of 50 AHF patients; despite an expected significant mortality rate (6% at 30 days and 19% at 6 months), the reduction of SMR severity was accompanied by a clinical improvement expressed as NYHA functional class, 6-min walk distance, LV volumes, and NT-proBNP plasma levels [24]. Further analysis found a positive impact of TMVr in terms of lower pulmonary artery pressures and resistances, with the consequence of including patients previously ineligible for this therapy on the HTx list (BTC) [25, 26]. The first results of the feasibility of a BTT strategy were described after the implantation of MitraClip alone or in combination with the Carillon device (Cardiac Dimensions, Kirkland, WA, USA) [27, 28]. The achievement of great clinical and hemodynamic benefits can even lead to recovery such as removing the patient from the HTx list (BTR) [29].

The “MitraBridge” study is an international, multicenter registry that collected data on MitraClip implantation in 119 AHF patients with severe or moderate-to-severe SMR [30]. The aim of the investigators was to analyze the outcomes of TMVr as a bridge strategy in this extreme setting of AHF patients. Baseline characteristics depict a clearly different population from those of published trials (80% of patients were formally ineligible according to the Clinical Outcomes Assessment of the MitraClip Percutaneous Therapy [COAPT] trial criteria) [31]: median age was 58 years, almost all of them (95%) were in NYHA class III − IV, severely depressed LV function (ejection fraction with a median of 26% and below 30% in 71.5% of patients), an abnormal cardiac index (median of 1.9 L/min/m^2^ and below 2 L/min/m^2^ in 60% of patients), and 40.5% of them had an INTERMACS profile of 1 to 4. Reported results showed that MitraClip implantation as a bridge strategy was safe with a procedural success of 87.5% and no deaths at 30 days. One-year outcomes found two-thirds of patients free from the composite endpoint (all-cause death, urgent HTx or LVAD implantation, first rehospitalization for heart failure), 15.5% became eligible for HTx (BTC), and nearly a quarter of them (27 patients) were removed from consideration for HTx because of clinical improvement (BTR). These exploratory results are promising and provide a proof of concept of MitraClip implantation in patients with AHF who are on the waiting list or at that moment not eligible or at high risk for HTx.

## Transcatheter mitral valve replacement

Transcatheter mitral valve replacement (TMVRpl) is a promising technology that can provide predictable and reproducible results. Currently, only limited specialized centers can perform this technique, whose evidence is still too scarce to be as widespread as TMVr.

**Fig. 3:**
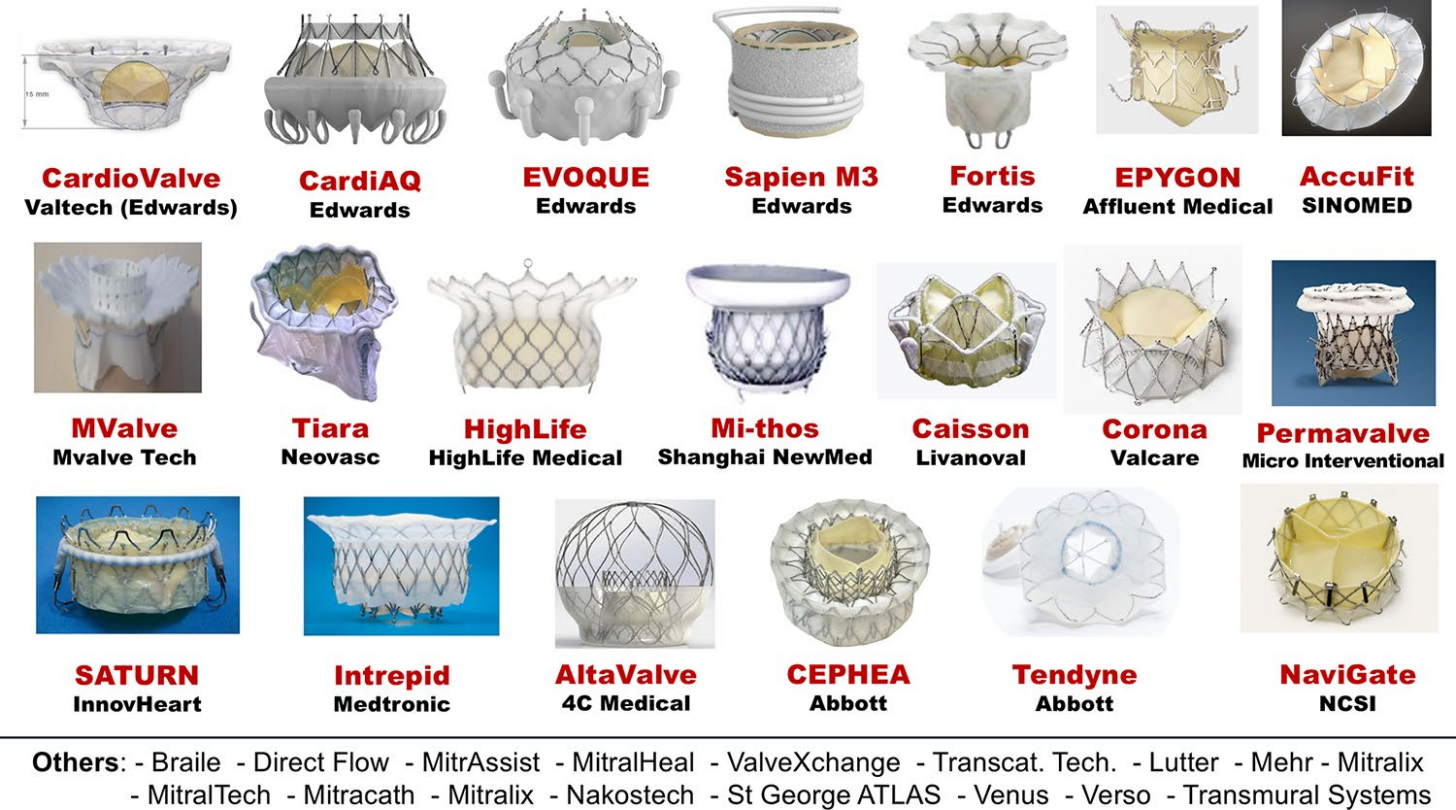
Transcatheter mitral valve replacement devices. Reproduced from Vessel Plus 2021;5:6 [20]

The available TMVRpl devices are trileaflet biological valves built with xenogenic leaflets that are mounted on self-expanding frames (Fig. 3) [20]. Transcatheter valve implantation on the mitral valve is not straightforward as its counterpart on the aortic valve due to complex mitral anatomy characterized by a peculiar D-shape and a well-represented sub-valvular apparatus. The conformation of the native valve hinders the generation of a uniform radial force with the risk of paravalvular leakage and prosthesis migration. The approach of some companies to hold the valve in place is the application of anchors to the annulus, the sub-valvular apparatus, or the left ventricular apex. The risk of paravalvular leakage may only be partially decreased with the over-expansion and circularization of the valve, because of the potential obstruction of the left ventricular outflow tract. This dreaded complication also occurs in the case of mitral aortic angle < 120°, severe LV septal hypertrophy, extremely wide annulus, and too ventricular implant sites. Most of the systems under investigation require transapical access that enables the delivery of large devices and allows for better coaxiality with the native valve.

### Clinical evidence

TMVRpl experience in native mitral valves is still limited to trials with slightly more than 500 procedures. The Tendyne (Abbott Vascular; Santa Clara, CA) has been the first transcatheter mitral valve implantation system to be approved in Europe in 2020 and, to date, no device has gained the Food and Drug Administration approval for the USA. The analysis of the first 308 TMVRpls shows that the procedure is feasible and, compared to the COAPT trial, provides better results in terms of post-procedural mitral regurgitation reduction (moderate or severe in 1.5% vs. 7.3%, respectively), symptomatic improvement (NYHA class III or IV 14.4% vs. 20.3%, respectively), and hospitalizations for heart failure (26.4% vs. 35.7%, respectively) [31, 32]. However, the rates of complications (stroke, endocarditis, and unplanned mitral valve surgery) and mortality (13.6% at 30 days, 27.6% at a mean follow-up of 10 months) are still unacceptable. It is necessary to specify that these data come from studies that investigated different TMVRpl systems and included very heterogeneous patients: from the elderly with a relevant comorbidity burden and very high operative risk (compassionate use) to those who were fitter and passed a selective enrollment screening. However, the improvements that have been achieved over time are promising. A real-world series of 11 patients treated with Tiara and Tendyne systems reported a 100% technical success with no procedural or in-hospital mortality [33]. Recent data on TMVRpl with new-generation devices who are currently under clinical evaluation (2 AltaValve, 5 CardioValve, 4 Cephea, 14 Evoque, 15 Highlife, 50 Interpid, 45 M3, 109 Tendyne, 79 Tiara) show a weighted mean technical success of 94%, post-procedural mitral valve mean gradient of 3.7 (3.5–3.8), at least moderate residual MR in 3.1% of cases, and procedural and 30-day mortality rates of 1.4% and 9.5%, respectively [34].

TMVRpl for severe SMR in patients at high surgical risk and previous mitral surgery (bioprosthesis or annuloplasty) or severe mitral annular calcification (valve-in-valve [ViV], valve-in-ring [ViR], and valve-in-MAC, respectively) can be performed with the transcatheter aortic SAPIEN XT and S3 (Edwards Lifesciences, Irvine, CA), the MyVal (Meril, Vapi, India), or the transcatheter pulmonary Melody (Medtronic, Minneapolis, MN) valve [35–37]. Data from the VIVID (Valve-in-Valve International Data) registry on mitral ViV and ViR procedures identify the SAPIEN 3 as the most frequently used device (~ 42%) and the transapical as an access route more commonly used in ViV than in ViR (64% vs. 51%). In terms of procedural results, MVARC-defined technical success ranges from 82 (ViR) to 93.5% (ViV) and MVARC-defined device success from 32% (ViR) to 39.4% (ViV). The most common cause of device failure can be identified in postprocedural mean gradient ≥ 5 mmHg (96% ViV, 88% ViR). In addition to lower success rates, ViR was burdened by higher rates of malposition, increased need for second transcatheter valve implantation, and higher rates of LVOT obstruction. These procedural outcomes are accompanied by a higher 1-year mortality for ViR (23.2% vs. 13.8%, ViR vs. ViV, respectively) that persists at 4 years [38]. The particularly rigid and non-circular nature of surgical rings may be the cause of worse outcomes due to more residual MR and device underexpansion.

A limitation to TMVRpl is represented by the transapical access: patients with a remodeled LV or with ischemic scars may not tolerate well this approach. As they are developed, systems that deliver the valve with a transseptal route through transfemoral introducers are expected to result in fewer complications and better outcomes.

Valve durability after TMVRpl is another great concern that needs to be better elucidated. The age of patients undergoing TMVRpl (about 10 years younger than recipients of aortic valves) plays an important role in the rate of structural valve degeneration [39]. Enhanced hemodynamic shear stress, the difference in calcium metabolism, patient prosthesis mismatch, and residual leaflet antigenicity expose young patients to an accelerated valve deterioration [40]. Another key factor of this process is represented by the systolic pressure gradient that produces greater mechanical stress on mitral bioprosthesis if compared to the aortic ones.

A relevant risk of valve thrombosis undermines TMVRpl: 6% to 8% after the implantation of Tendyne (Abbott Vascular, Abbott Park, IL), Fortis (Edwards Lifesciences, Irvine, CA), and HighLife (HighLife Medical, Irvine, CA) device [41–43]. The adoption of oral anticoagulation in addition to antiplatelet therapy resulted in no thrombotic complications at 1 year, at the expense of a significant rate (18%) of 30-day major bleedings after intrepid valve (Medtronic Inc., Redwood City, CA) implantation [44]. Taking the cue from the recommendations after surgical mitral valve replacement, an anticoagulation strategy for the first 3 months seems reasonable to protect against early valve thrombosis [19]. After this period, when endothelization is complete, a serial clinical and imaging follow-up should guide toward a prompt diagnosis and treatment of any late thrombotic complication.

## Future perspectives

At present, there are limited exploratory studies on the transcatheter treatment of SMR in patients with AHF. While a direct prognostic improvement after correction of SMR has yet to be proved, AHF patients can greatly benefit from the following: hemodynamic stabilization, symptomatic relief, normalization of pulmonary arterial pressures, and reduction in hospitalizations for acute heart failure. Obtaining these results may lead to the clinical consequences of reaching the HTx in good enough clinical status (BTT), becoming eligible for the HTx (BTC), and being delisted for clinical improvement (BTR). Therefore, achieving “not-so-hard endpoints” in patients with AHF and SMR can translate into great clinical implications.

More studies are needed to investigate who more likely will benefit the most from SMR treatment at this advanced stage of LV disease. Since left and right ventricular reverse remodeling have been found as turning points of favorable outcomes, it should be assessed whether this phenomenon can also occur in this population [45, 46].

The lack of sufficient evidence and the lesson learned from the surgical experience make TMVr preferable over TMVRpl [21]. However, as more studies on TMVRpl are completed, this technology could gain much appeal and be considered a viable alternative to TMVr instead of being regarded as a second choice. TMVRpl devices can provide more predictable results in terms of MR reduction, require a less-technically demanding implantation, and have the potential to be able to adapt to such different anatomies, including those that represent exclusion criteria for TMVr (multisegment or commissural disease, perforations, and clefts).

## Conclusions

Transcatheter therapies for SMR may play an important role in AHF patients. Exploratory studies proved the procedural safety and clinical efficacy (BTC, BTT, and BTR) of this treatment. Further investigations are needed to better characterize AHF patients with concomitant SMR, identify optimal candidates for transcatheter therapies, and understand the consequences of the different bridge strategies. While TMVr has more evidence in this setting, TMVRpl is expected to become a valid alternative in the near future. Having a complete mitral toolbox will be the key to handle all the anatomies and provide a patient-tailored approach.
